# Air Pollution: Floral Scents Going Off the Air?

**DOI:** 10.1289/ehp.116-a334

**Published:** 2008-08

**Authors:** Carol Potera

Would a flower by any other name always smell as sweet? Maybe not, if air pollution has anything to do with it. Researchers at the University of Virginia in Charlottesville report that three common constituents of smog destroy floral scents released by flowers to attract bees and other pollinators. In fact, flower scents traveled four times farther in the 1840s, when European scientists first began documenting ozone pollution, than they do under today’s air conditions, according to modeled simulations run by the researchers. Because pollinating insects rely partly on scents to find flowers, the loss of fragrant plumes could make it harder for insects to locate pollen sources, jeopardizing pollinators and crops alike. The researchers are beginning field trials this month to see if real-world tracking of airborne floral scents match the results predicted by the model.

The vitality of plants depends on pollination, according to principal investigator Jose Fuentes, a meteorologist. Additionally, if pollinators are forced to spend more time foraging for food yet collect less pollen to feed their young, insect colonies may suffer nutritionally. Both problems could impact our food supply. “We need to preserve pollinators because they provide useful services,” Fuentes says.

It had already been established that when fragrance molecules wafting downwind meet up with air pollutants, chemical reactions alter the floral scents and contribute to production of compounds such as acetone, formaldehyde, and carbon monoxide. In the current study, Fuentes and colleagues Quinn McFrederick and James Kathilankal estimated the fate of three common volatile hydrocarbons emitted by flowers as they encountered increasing levels of ozone, hydroxyl radicals, and nitrate radicals. In the summer of 2002, the researchers measured temperature, wind turbulence, and other factors that determine the rate of floral scent emission and movement at an experimental farm in Virginia. They observed how these factors affected the release of scent from a plot of snapdragons that grew wild on the farm.

The researchers plugged these data into a model to test different air pollution scenarios ranging from conditions that prevailed during the 1840s to current summertime conditions in large eastern U.S. cities, where ozone levels can exceed 120 ppb by volume. Under 1840s conditions, only 20% of scents were altered by chemical reactions within a 1,000-meter radius downwind of the floral source. However, even slight elevations in pollutants—comparable to air quality today in rural areas with little industrial emissions—altered more than 40% of the scents within a 500-meter radius of the floral source. In the most polluted scenario, only 25% of the scents survived 300 meters downwind. Fuentes and colleagues reported the findings in volume 42, issue 10 (March 2008) of *Atmospheric Environment*.

Do compounds generated when floral scents are chemically altered actually worsen air pollution? “We haven’t thought about this in [terms of] air quality,” Fuentes says. He says the findings also raise special concerns about the fate of nighttime pollinators such as moths, which rely largely on scent to find flowers (in contrast to bees, which use both color and scent during daytime).

Fuentes cautions against applying the new findings to colony collapse disorder (CCD), an unexplained phenomenon that has decimated bee colonies in the past two years. He says some of the best evidence to date suggests CCD is more directly related to infectious agents and pesticides. However, he says, any effects from air pollution “are probably an added stress that bees have to cope with.”

Other insect behaviors that orient by chemical scents, such as beetles mating, also could be disturbed by air pollution. On the other hand, some plants may benefit from disrupted airborne signals: “If insects can’t smell plants, they can’t come to eat them,” says Jay Evans, a research entomologist at the U.S. Department of Agriculture, who calls the Fuentes study “a beautiful mix of good ecology and chemistry.”

Pollinators aren’t the only species dealing with the olfactory effects of air pollution. In the January 2006 issue of *Chemical Senses*, Robyn Hudson and colleagues reported that residents of Mexico City, which has some of the world’s worst air pollution, were significantly less able to detect and distinguish between food odors than were residents of Tlaxcala, a geographically similar area with much lower air pollution. The difference was observed in multiple age groups, even when smokers were removed from the analysis, strongly suggesting a link with pollutants that may damage the olfactory epithelium.

## Figures and Tables

**Figure:**
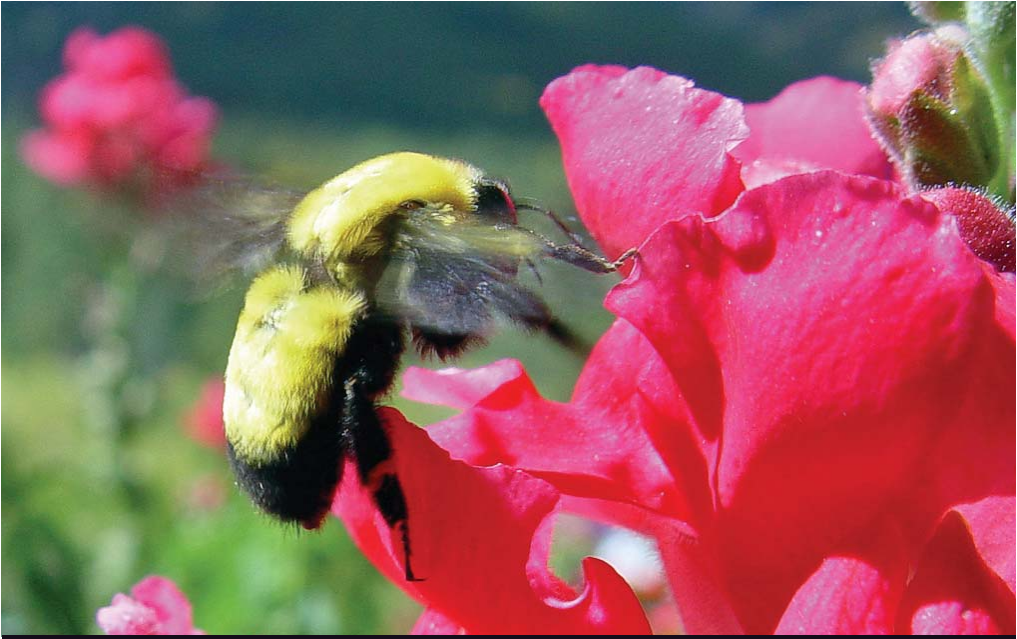
Air pollutants interfere with pollinators’ ability to locate flowers by scent

